# Positron Emission Tomography Molecular Imaging Biomarkers for Amyotrophic Lateral Sclerosis

**DOI:** 10.3389/fneur.2019.00135

**Published:** 2019-03-01

**Authors:** Sheena Chew, Nazem Atassi

**Affiliations:** Department of Neurology, Harvard Medical School, Neurological Clinical Research Institute, Massachusetts General Hospital, Boston, MA, United States

**Keywords:** amyotrophic lateral sclerosis, neuroimaging, positron emission tomography, biomarker, diagnostic biomarker, pharmacodynamic biomarker, therapeutic development

## Abstract

Amyotrophic lateral sclerosis (ALS) is a fatal neurodegenerative disorder with limited treatment options. Despite decades of therapeutic development, only two modestly efficacious disease-modifying drugs—riluzole and edaravone—are available to ALS patients. Biomarkers that can facilitate ALS diagnosis, aid in prognosis, and measure drug pharmacodynamics are needed to accelerate therapeutic development for patients with ALS. Positron emission tomography (PET) imaging has promise as a biomarker for ALS because it permits visualization of central nervous system (CNS) pathology in individuals living with ALS. The availability of PET radioligands that target a variety of potential pathophysiological mechanisms—including cerebral metabolism, neuroinflammation, neuronal dysfunction, and oxidative stress—has enabled dynamic interrogation of molecular changes in ALS, in both natural history studies and human clinical trials. PET imaging has potential as a diagnostic biomarker that can establish upper motor neuron (UMN) pathology in ALS patients without overt UMN symptoms, as a prognostic biomarker that might help stratify patients for clinical trials, and as a pharmacodynamic biomarker that measures the biological effect of investigational drugs in the brain and spinal cord. In this Review, we discuss progress made with 30 years of PET imaging studies in ALS and consider future research needed to establish PET imaging biomarkers for ALS therapeutic development.

## Introduction

Amyotrophic lateral sclerosis (ALS) is a devastating disorder characterized by degeneration of motor neurons in the brain and spinal cord. It is clinically heterogeneous and shares clinical and pathological features with frontotemporal dementia (FTD). ALS invariably leads to weakness and death; ~70–80% of ALS patients die within 5 years of symptom onset (1).

Riluzole and edaravone are currently the only disease-modifying treatments for ALS. More efficacious therapy is urgently needed. Fortunately, the recent expansion of knowledge about genetics and pathophysiology of ALS (2) has generated a large pipeline of potential therapeutic agents to be tested in ALS. Biomarkers for ALS are now urgently needed to stratify patients for trial enrollment, to demonstrate biological drug effect, and to guide dose-selection and go-no-go decisions in early phase clinical trials.

Multiple types of biomarkers are being developed for use in ALS (3–5). Electrophysiological biomarkers of the upper motor neurons (UMNs) [transcranial magnetic stimulation (6)] and lower motor neurons (LMN) [motor unit number index (7) and electrical impedance myography (8)] directly quantify physiology of diseased tissues. Biological fluid-based biomarkers such as phosphorylated neurofilament heavy chain in cerebrospinal fluid (CSF) (9, 10), neurofilament light chain from CSF or serum (10–14), and urine p75 neurotrophin receptor extracellular domain (15) are being evaluated as markers of neuronal degeneration. Neuroimaging biomarkers using magnetic resonance imaging (MRI) or positron emission tomography (PET) techniques can objectively visualize changes associated with the disease processes and help to understand the mechanisms of neurodegeneration *in vivo* (16).

This Review will focus on development of PET molecular imaging biomarkers for ALS. References for this Review were identified by searching PubMed for the terms “amyotrophic lateral sclerosis” or “ALS” or “motor neuron disease” or “MND” AND “PET” or “positron emission tomography.” As of October 11, 2018, 222 articles were identified. We excluded articles that were not focused on motor neuron diseases (17), were animal or post-mortem studies (18), were not focused on PET imaging (19), were not dedicated to brain or spinal cord (2), were not written in English (12), were inaccessible (7), studied fewer than 5 ALS or MND cases (20), or were literature reviews or guidelines (21), resulting in 48 papers.

## The Development of PET Imaging in ALS

PET imaging uses positron-emitting radioisotopes that are incorporated into molecules of interest (“tracers”), which are injected intravenously and enter the central nervous system (CNS). When positrons encounter electrons, they annihilate and emit pairs of gamma rays that travel away from one another at a 180° angle. The detection of gamma ray pairs by the PET camera enables localization of the annihilation event and subsequent three-dimensional reconstruction of radiotracer distribution in the tissue of interest (16). The development of PET tracers that permit visualization of glucose metabolism, cerebral blood flow, neurotransmitter metabolism, neuroreceptor binding, inflammation, and oxidative stress have permitted a deep investigation into the molecular pathophysiology of ALS *in vivo* (Table 1).

**Table 1:** PET studies in ALS.

**References**	**Tracer**	**Target(s)**	**Cross sectional results**	**Longitudinal results**	**Clinical correlation**
**GLUCOSE METABOLISM**					
Dalakas et al. (18)	^18^F-FDG	Glucose metabolism	12 ALS vs. 11 HC: diffuse hypometabolism in cortex and basal ganglia of ALS patients with UMN involvement. Cerebellar metabolism similar between ALS and HC.	4 ALS with 2+ scans; variable changes in metabolism over time	No statistically significant difference in cortical metabolism between ALS patients without UMN signs and HC.
Hatazawa et al. (22)	^18^F-FDG	Glucose metabolism	12 ALS vs. 11 HC: diffuse hypometabolism, greatest in motor-sensory cortex and putamen. No difference in metabolism in patients without UMN involvement	4 ALS with repeat studies showed reduction in metabolism over time	Cortical hypometabolism associated with disease duration at time of scan.
Ludolph et al. (23)	^18^F-FDG	Glucose metabolism	18 ALS vs. 12 HC: diffuse hypometabolism in frontal regions not reaching statistical significance	None	Hypometabolism in frontal regions correlates with frontal dysfunction measured by neuropsychologic testing. No correlation between hypometabolism and disease duration at time of scan.
Hoffman et al. (24)	^18^F-FDG	Glucose metabolism	7 ALS vs. 11 HC: no statistically significant difference when corrected for multiple comparisons	3 ALS with repeated scans after 1 year; no significant reduction in uptake despite clinical progression	Decreased motor strength correlated with hypometabolism in precentral gyri and hypermetabolism in middle frontal gyrus
Garraux et al. (25)	^18^F-FDG	Glucose metabolism	3 ALS-FTD vs. 46 HC, 10 FTD vs. 46 HC: frontal and anterior temporal hypometabolism	None	No statistically significant differences in cortical hypometabolism between 3 ALS-FTD patients and 10 FTD patients when corrected for multiple comparisons
Jeong et al. (26)	^18^F-FDG	Glucose metabolism	8 ALS-FTD vs. 11 HC: hypometabolism in bilateral frontal lobes, basal ganglia, thalamus	None	No statistically significant differences in cortical metabolism between 8 ALS-FTD and 29 FTD patients
Renard et al. (19)	^18^F-FDG	Glucose metabolism	4 ALS-FTD vs. 6 ALS	None	ALS patients with FTD had hypometabolism in dorsolateral prefrontal, medial/lateral premotor cortices, insular cortices, anterior temporal lobes compared to ALS patients without FTD
Boeve et al. (27)	^18^F-FDG	Glucose metabolism	5 C9 ALS: in 4 of 5, hypometabolism in anterior cingulate, frontal cortices compared to age-segmented normative database	1 ALS with second scan after 2 years showing more prominent cortical hypometabolism	Frontal cortical and anterior cingulate hypometabolism correlated with poor performance on neuropsychological measures of psychomotor speed, word fluency, sustained attention
Cistaro et al. (28)	^18^F-FDG	Glucose metabolism	32 ALS vs. 22 HC: Hypermetabolism in amygdala, midbrain, pons, cerebellum.	None	13 bulbar onset vs. 19 spinal onset ALS: relative hypometabolism in bilateral frontal cortex, right insula, anterior cingulate, precuneus, interior parietal lobe. Bulbar onset patients with lower neuropsychological scores in verbal fluency
Lai et al. (29)	^18^F-FDG	Glucose metabolism	10 spinobulbar muscular atrophy vs. 5 HC: hypometabolism in frontal areas	None	None reported
Clark et al. (30)	^18^F-FDG	Glucose metabolism	9 Primary spastic dysarthria vs. HC: variable degrees of hypometabolism in premotor and motor cortices	None	Hypometabolism in premotor and motor cortices associated with symptom duration >2 years
Pagani et al. (31)	^18^F-FDG	Glucose metabolism	195 ALS vs. 40 HC: Hypometabolism in frontal, premotor, occipital cortices. Hypermetabolism in midbrain, temporal pole, hippocampus	None	Bulbar onset ALS patients had more rostral pattern of hypometabolism compared to spinal onset ALS patients. Analysis of Brodmann areas 6, 7, 9-11, 13, 17, 18, 21, 22, 24, 32, 37-40, 47 discriminated ALS from HC scans with 95.4% sensitivity and 82.5% specificity
Van Laere et al. (32)	^18^F-FDG	Glucose metabolism	59 sALS vs. 20 HC: Hypometabolism in premotor and frontal cortices. Hypermetabolism in hippocampus, amygdala, brainstem, occipital, cerebellum. Similar pattern between 59 sALS, 7 PLS and 11 C9 ALS	None	Severe hypometabolism in frontotemporal regions correlated with shorter survival. Prefrontal hypometabolism is correlated with lower ALSFRS-R scores. Support vector machine analysis discriminated ALS from HC scans with 95.8% sensitivity, 80% specificity; PLS from HC with 57.1% sensitivity, 100% specificity.
Cistaro et al. (33)	^18^F-FDG	Glucose metabolism	15 C9 ALS vs. 30 sALS: hypometabolism in cingulate, insula, caudate, thalamus, left frontal and superior temporal cortex. Hypermetabolism in midbrain, occipital cortex, globus pallidus, left inferior temporal cortex. 12 sALS-FTD vs. 30 sALS: hypometabolism in orbitofrontal, prefrontal, anterior cingulate, insula. Hypermetabolism in occipital, left precentral/postcentral, superior temporal cortices. 15 C9ALS vs. 12 sALS-FTD: hypometabolism in left temporal cortex.	None	Genotype-phenotype correlation: widespread cortical hypometabolism in C9 ALS more reminiscent of sALS-FTD than sALS, despite lack of FTD diagnosis in C9 patients.
Rajagopalan and Pioro (34)	^18^F-FDG	Glucose metabolism	18 ALS-FTD vs. 15 HC: hypometabolism in frontotemporal lobes, cingulum, cerebellum, and motor cortex when normalized against pons and whole-brain. Most areas of hypometabolism corresponded with areas of gray matter atrophy.	None	None reported
Canosa et al. (35)	^18^F-FDG	Glucose metabolism	20 ALS-FTD vs. 150 ALS (94 cognitively normal, 37 with cognitive impairment, 9 with behavioral impairment, 10 with nonspecific impairment): hypometabolism in frontal and prefrontal regions.	None	Continuum of frontal lobe hypometabolism correlates with continuum of cognitive impairment
Van Weehaeghe et al. (17)	^18^F-FDG	Glucose metabolism	70 ALS (training set), 105 ALS (validation set) vs. 20 HC (used for both training and validation set): hypometabolism in frontal, premotor, inferolateral, parietal cortices. Hypermetabolism in primary visual cortex, cerebellum, upper brainstem, medial temporal cortex. 10 PLS vs. 20HC with similar pattern. Training and validation ALS cohorts had identical hypo- and hyper-metabolism patterns when compared to HC.	None	Frontotemporal hypometabolism predictive of shorter survival. Using volume of interest (VOI)-based discriminant analysis of training set: 88.8% accuracy in classifying ALS or PLS vs. HC in 105 prospective validation cases, if PMA scans excluded. Using voxel-based support vector machine (SVM) approach: 100% accuracy for classifying ALS or PLS vs. HC, if PMA scans excluded.
Marini et al. (36)	^18^F-FDG	Glucose metabolism	30 ALS vs. 30 HC: hypermetabolism in spinal cord	None	Spinal hypermetabolism (>5th decile) associated with higher mortality rate at 3 years
Matias-Guiu et al. (20)	^18^F-FDG ^18^F-florbetaben	Glucose metabolism, amyloid deposition	18 ALS vs. 24 HC: hypometabolism in frontal area, hypermetabolism in cerebellum. Concurrent use of tracer ^18^F-florbetaben showed no significant difference in amyloid uptake between ALS and HC.	None	Cognitive impairment associated with decreased frontoparietal metabolism
Buhour et al. (37)	^18^F-FDG	Glucose metabolism	37 ALS vs. 37 HC: hypometabolism in right paracentral lobule, left inferior parietal gyrus, bilateral thalamus, left superior medial frontal gyrus, cerebellar vermis. Hypermetabolism cerebellar lobules, medial temporal cortex, fusiform cortex.	None	Hypometabolism in hippocampus negatively correlated with changes in memory. Hypometabolism in left fusiform gyrus negatively correlated with theory of mind
Yamashita et al. (38)	^18^F-FDG ^11^C-flumazenil	Glucose metabolism, blood flow measured by early flumazenil binding	10 ALS vs. 10 HC: hypermetabolism in spinal cord ipsilateral to weakness at C5 and T1. No difference in flumazenil in spinal cord. Concurrent use of tracer ^11^C-flumazenil showed no difference in spinal cord uptake between ALS and HC.	None	Cervical hypermetabolism associated with ipsilateral arm weakness
D'Hulst et al. (39)	^18^F-FDG	Glucose metabolism	ALS (175 training scans from Belgium, 195 validation scans from Italy): minor differences in metabolism between ALS groups across two centers. HC (20 training scans from Belgium, 40 validation scans from Italy): prefrontal hypometabolism in Italian HC compared to Belgian HC cohort. Italian HC scans from patients with lung malignancy (no neurologic disease) who underwent oncologic surveillance PET scans	None	Using SVM analysis of training set, classified ALS or HC from validation set with 95% sensitivity, 12% specificity. Unable to reverse analysis using validation cohort as training cohort and vice versa. Diagnostic algorithm to classify ALS from control scans was unsuccessful when control scans came from patients with non-neurologic illness rather than healthy volunteers
**CEREBRAL BLOOD FLOW**					
Kew et al. (40)	C^15^O_2_	Regional CBF	12 ALS vs. 6 HC: At rest, decreased CBF in sensory and motor cortex, supplementary motor area, parietal regions. With joystick movement task, increased CBF in contralateral motor cortex and adjacent premotor and parietal areas	None	In ALS, poorer verbal fluency associated with decreased CBF in right parahippocampus, bilateral anterior thalamus, right anterior cingulate during joystick movement task No correlation between verbal fluency and resting CBF
Kew et al. (41)	C^15^O_2_	Regional CBF	10 ALS vs. 5 HC: decreased CBF during joystick movement task	None	In ALS, poorer verbal fluency associated with decreased CBF in right parahippocampus, anterior thalamus, anterior cingulate during task
Tanaka et al. (42)	C^15^O_2_^15^O_2_	Regional CBF	9 ALS vs. 13 HC: non-significant reductions in CBF and oxygen metabolism. 4 ALS with dementia vs. 13 HC: decreased CBF and metabolism in anterior cerebral hemispheres and cerebellum	None	Comparison of CBF between ALS with and without clinical dementia not reported
Abrahams et al. (43)	C^15^O_2_	Regional CBF	6 ALS vs. 6 HC: decreased activation (smaller increase in CBF compared to CBF in control condition) during word generation task in right dorsal prefrontal, bilateral inferior parietal lobule, left middle/superior temporal gyri 6 ALS with cognitive impairment vs. 6 HC: decreased activation during word generation task in bilateral dorsolateral prefrontal cortex, medial pre-frontal, premotor, anterior thalamic, insular cortex	None	Poor verbal fluency associated with decreased activation in bilateral prefrontal, premotor, insular cortices, thalamus
**NEUROINFLAMMATION**					
Turner et al. (44)	^11^C-PK11195	TPSO	10 ALS vs. 14 HC: increased uptake in precentral gyri, pons, thalamus, dorsolateral prefrontal cortices	None	Increased uptake correlated with UMN-B. No correlation in ALSFRS-R or disease duration.
Johansson et al. (45)	^11^C-L-deprenyl-D2	MAO-B—postulated nonspecific measure of astrocytosis	7 ALS vs. 7 HC: increased binding rate in white matter and pons, decreased binding rate in parietal and temporal cortices	2 ALS scans at 8 and 10 months, no change	No statistically significant correlation between binding and clinical characteristics
Corcia et al. (21)	^18^F-DPA-713	TPSO	10 ALS vs. 8 HC: increased uptake in primary motor, supplementary motor, and temporal cortex. No increased activation in pons of bulbar-onset ALS patients.	None	No correlation between uptake and age, disease duration, or ALSFRS-R
Zurcher et al. (46)	^11^C-PBR28	TPSO	10 ALS vs. 10 HC: increased uptake in motor cortices and corticospinal tracts	None	Increased uptake correlated negatively with ALSFRS-R, positively with UMN-B score.
Alshikho et al. (47)	^11^C-PBR28	TSPO	10 ALS vs. 10 HC: increased uptake in left motor cortex correlates with decreased cortical thickness and fractional anisotropy	None	Increased uptake correlated positively with UMN-B score.
Paganoni et al. (48)	^11^C-PBR28	TSPO	10 PLS vs. 10 HC: increased uptake in anatomically relevant motor regions co-localized with regional gray matter atrophy and decreased subcortical fractional anisotropy	None	No correlation between uptake and UMNB and ALSFRS-R
Albrecht et al. (49)	^11^C-PBR28	TSPO	10 ALS, 10 HC, 10 low back pain. Occipital cortex may serve as pseudoreference region rather than whole brain for measuring PBR28 uptake.	None	None reported
Alshikho et al. (50)	^11^C-PBR28	TSPO	53 ALS vs. 21 HC: increased uptake in precentral and paracentral gyri. 11 PLS vs. 21 HC: increased uptake in subcortical white matter of same regions. Increased uptake colocalizes with cortical thinning, reduced frational anisotropy, increased mean diffusivity.	10 scans 6 months apart, no significant change despite decrease in ALSFRS-R by 3 points	Increased uptake in regions of interest correlated positively with UMNB score. Uptake did not change significantly despite clinical decline
Ratai et al. (51)	^11^C-PBR28	TSPO	40 ALS: PBR28 uptake correlates positively with mI/Cr and negatively with NAA/Cr in precentral gyri.	None	ALSFRS-R score correlated positively with NAA/Cr and negatively with mI/Cr. UMNB score correlated positively with PBR28 uptake and mI/Cr, negatively with NAA/Cr
**GABAergic FUNCTION**					
Lloyd et al. (52)	^11^C-flumazenil	GABAa receptor	17 ALS vs. 17 HC: decreased uptake in bilateral prefrontal, parietal, visual association, left premotor/motor cortex.	None	No differences in uptake between ALS patients with or without pseudobulbar affect
Turner et al. (53, 54)	^11^C-flumazenil	GABAa receptor	24 sALS vs. 24 HC: decreased uptake in premotor, motor, posterior association regions. 10 SOD1 D90A ALS vs. 24 HC: decreased uptake in left frontotemporal junction, anterior cingulate. 2 pre-symptomatic SOD1 D90A—decreased uptake in left frontotemporal junction. 4 PLS vs. HC: relative preservation of anterior and orbitofrontal binding compared to ALS.	None	In sALS, decreased uptake in dominant hemisphere correlated with higher UMN-B score. No correlation between uptake and ALSFRS-R. In SOD1 D90A ALS, uptake correlated positively with ALSFRS-R rather than UMNB.
Wicks et al. (55)	^11^C-flumazenil	GABAa receptor	12 ALS with cognitive testing	None	Correlation between poorer performance in verbal fluency and reduced binding in right inferior frontal gyrus, superior temporal gyrus, anterior insula.
					Correlation between poorer confrontation naming and reduced binding in left inferior frontal gyrus/middle frontal gyrus.
Yabe et al. (56)	^11^C-flumazenil	GABAa receptor	10 ALS with cognitive testing	None	Correlation between writing errors and reduced binding in bilateral anterior cingulate gyrus
**SEROTONERGIC FUNCTION**					
Turner et al. (57)	^11^C-WAY100635	5-HT1a receptor	21 ALS vs. 19 HC: marked decreased global cortical binding (21%). Regional decreased binding in frontotemporal regions, cingulate, lateral precentral, parahippocampal, and fusiform gyri	None	Greater decrease in cortical binding in ALS (21%) compared to historical data in depression (12%) and Parkinson's (15%). Trend toward greater reductions in binding in patients with bulbar involvement
Turner et al. (58)	^11^C-WAY100635	5-HT1a receptor	11 SOD1 D90A ALS vs. 19 HC: decreased global cortical binding (12%), less dramatic when compared with reduction in binding in sporadic ALS vs. HC (21%)	None	Less reduction in cortical binding of D90A ALS compared to sporadic ALS, despite lower ALSFRS-R scores
**DOPAMINERGIC FUNCTION**					
Takahashi et al. (59)	^18^F-6-fluorodopa	Levodopa metabolism	16 ALS vs. 13 HC: no difference in mean striatal uptake	None	Negative correlation between 6-fluorodopa uptake and duration of ALS symptoms. No correlation between uptake and severity of symptoms.
Przedborski et al. (60)	^18^F-6-fluorodopa	Levodopa metabolism	7 SOD1 familial ALS, 7 non-SOD1 familial ALS, 14 HC. 5/14 familial ALS with reduced uptake in nigrostriatal region, more commonly seen in non-SOD1 patients.	None	No correlation between binding and duration of symptoms
Hideyama et al. (61)	^18^F-6-fluorodopa, ^11^C-N-methylspiperone	Levodopa metabolism and D2/D3 receptor antagonist	5 ALS with clinical parkinsonism: preganglionic and postganglionic striatonigral dopaminergic systems preserved	None	Parkinsonism in ALS patients not correlated with striatonigral dysfunction
Fu et al. (62)	^18^F-fallypride	D2/D3 receptor antagonist	17 ALS vs. 11 HC: decreased binding in bilateral nucleus accumbens, frontal lobes, superior frontal gyri, left temporal lobe, left angular gyrus. No difference in striatum.	None	None reported
**OXIDATIVE STRESS**					
Ikawa et al. (63)	^62^Cu-ATSM	Intracellular reductive state	12 ALS vs. 9 HC: increased uptake in bilateral pre- and post- central gyri and paracentral lobule, right superior parietal lobule.	None	Increased uptake negatively correlated with ALSFRS-R. No correlation between uptake and disease duration

*ALS, amyotrophic lateral sclerosis; ALSFRS-R, ALS functional rating scale-Revised; C9, C9orf72 hexanucleotide repeat expansion; CBF, cerebral blood flow; FTD, frontotemporal dementia; HC, healthy control; mI/Cr, myoinositol/creatine ratio; NAA/Cr, N-acetylaspartate/creatine ratio; PMA, primary muscular atrophy; sALS, sporadic ALS; SOD1, superoxide dismutase 1; TSPO, translocator protein; UMN, upper motor neuron; UMN-B, Upper Motor Neuron Burden scale*.

### Glucose Metabolism and Cerebral Blood Flow

The first PET study in ALS, conducted in 1987, used the tracer ^18^F-fluorodeoxyglucose ([^18^F]-FDG) to demonstrate that ALS patients with UMN involvement had diffuse cortical hypometabolism compared to healthy controls (18). Subsequent [^18^F]-FDG PET studies found variable cortical hypometabolism in ALS (22–24). PET studies using radiolabeled carbon dioxide (C[^15^O]_2_), which detects alterations in regional cerebral blood flow (40–42), revealed decreased cerebral blood flow to the prefrontal cortex (41–43) and thalamus (41, 43) that correlated with cognitive impairment in ALS. These early PET findings suggested that ALS pathology expanded outside the motor cortex, years before ALS was widely accepted as a disorder on the same spectrum as frontotemporal dementia (FTD).

The 2011 discovery that C9orf72 hexanucleotide repeat expansions cause both ALS and FTD (64–66) motivated new [^18^F]-FDG PET studies that explored genotype-phenotype correlations and cognition in ALS. One study suggested that ALS patients with C9orf72 expansions had more widespread cortical hypometabolism than sporadic ALS patients (33), though this finding was not replicated (32). Other studies demonstrated frontal and prefrontal hypometabolism in patients with sporadic ALS-FTD compared to ALS patients without FTD (33–35).

In recent years, large cross-sectional [^18^F]-FDG PET studies have established that sporadic ALS is associated with hypometabolism in the premotor and frontal cortices and hypermetabolism in the brainstem (28, 31, 32). There is now interest in spinal cord imaging: two [^18^F]-FDG PET studies demonstrated hypermetabolism in the cervical cords of ALS patients (36, 38). These findings suggest potential differences between cortical vs. brainstem and spinal cord metabolism that warrant further exploration.

### Neuroinflammation

Neuroinflammation, specifically microglial activation, is a pathological hallmark of ALS (67, 68) and is associated with rate of disease progression (69). The 18 kD translocator protein (TSPO) is highly expressed on activated microglia and astrocytes (70, 71). Radiotracers that bind to TSPO thus can visualize neuroinflammation and gliosis *in vivo*. Indeed, early PET studies of neuroinflammation in ALS used the first-generation TSPO ligands [^11^C]-PK11195 (44) and [^18^F]-DPA-714 (21) to demonstrate the presence of widespread glial activation in brains of ALS patients compared to healthy controls.

The second-generation TSPO tracer [^11^C]-PBR28, which binds TSPO with an 80-fold higher specificity than [^11^C]-PK11195 (72), has enabled more precise PET evaluation of glial activation. Several [^11^C]-PBR28 PET studies demonstrated increased tracer uptake isolated to the motor cortices of ALS patients compared with controls (46, 47, 50). Areas of increased uptake correlated positively with Upper Motor Neuron Burden Scale and negatively with ALS Functional Rating Scale-Revised (ALSFRS-R) scores (46, 47, 50). Integrated [^11^C]-PBR28 PET and MRI scans established that areas of increased uptake co-localize with areas of cortical thinning and reduced fractional anisotropy (47, 50).

[^11^C]-PBR28 PET studies in patients with primary lateral sclerosis (PLS) found a pattern of glial activation similar to that seen in ALS patients, though tracer uptake was greatest in subcortical white matter in PLS patients and in cortical gray matter in ALS patients (48). The differences between ALS and PLS scans highlight the increased specificity of [^11^C]-PBR28 tracer and merit further investigation into why such differences in glial activation might exist in these two conditions.

In the largest longitudinal ALS PET study to date, 10 patients underwent [^11^C]-PBR28 PET scans twice over 6 months. Tracer uptake remained stable despite disease progression, as measured by a 3-point decrease in ALSFRS-R (50). This stability may mirror the pattern of beta-amyloid brain deposition in Alzheimer's disease, as measured by Pittsburg compound B (PiB) PET imaging: PiB uptake rises in patients developing mild cognitive impairment, then plateaus upon development of Alzheimer's dementia (73). Alternatively, it may reflect a bias toward recruitment of slowly-progressive patients into longitudinal neuroimaging studies. Longitudinal studies with larger sample sizes, rapidly progressing patients, and patients early in the disease course are needed to determine the natural history of glial activation in ALS.

### GABAergic Function

Cortical excitability is altered in ALS (6). To evaluate whether loss of GABAergic inhibition contributes to cortical hyperexcitability in ALS, PET studies were conducted using the GABA_A_ receptor ligand [^11^C]-Flumazenil. These studies showed widespread reductions in binding in ALS patients compared to controls (52), and found that reduced binding in the frontal lobes (55) and anterior cingulate gyri (56) in ALS patients correlated with poorer performance on language tasks. Additionally, patients with slowly progressive ALS caused by SOD1 D90A mutations had smaller reductions in binding compared to sporadic ALS patients (53). Taken together, these findings could suggest that loss of GABAergic cortical inhibition is part of ALS pathogenesis, though it is also possible that it reflects generalized cortical neuronal loss rather than specific loss of GABAergic inhibition.

### Serotonergic Function

The serotonin 5-hydroxytryptamine (5-HT1a) receptor is expressed widely in the cortex, including on layer III and V pyramidal neurons in the cortex (74). A PET imaging study using the 5-HT1a ligand [^11^C]-WAY100635 demonstrated decreased tracer binding in the frontotemporal regions, precentral, cingulate, parahippocampal, and fusiform gyri in non-depressed ALS patients compared to healthy controls (57). A follow up study reported smaller reductions in [^11^C]-WAY100635 uptake in patients with slowly progressive SOD1 D90A genetic ALS compared to sporadic ALS (58). Like the studies using GABA_A_ ligands, these studies suggest widespread neuronal loss or dysfunction in ALS patients that is less apparent in slowly progressive disease.

### Dopaminergic Function

Evidence of extramotor involvement in ALS has raised questions about its overlap with neurodegenerative disorders such as Parkinson's disease. Rare patients with ALS have parkinsonism, and post-mortem evaluation has revealed degeneration of the substantia nigra in ALS (75). To evaluate whether dopaminergic dysfunction plays a role in ALS pathogenesis, several PET studies were conducted using ligands that interrogate levodopa metabolism [[^18^F]-fluorodopa (59, 61)], bind to dopamine receptors in the striatum [[^11^C]-N-methylspiperone (61)], and bind to dopamine receptors in the cortex [[^18^F]-fallypride (62)]. The [^18^F]-fluorodopa and [^11^C]-N-methylspiperone studies showed no significant difference in levodopa metabolism or dopamine receptor binding in the striatum of ALS vs. control subjects, even in patients with overt parkinsonism (59, 61). Conversely, the [^18^F]-fallypride PET study showed decreased dopamine binding in the cortex of ALS patients (62), even though the patients were not noted to have clinical parkinsonism. One interpretation of these studies is that ALS is associated with cortical rather than striatal dopaminergic dysfunction. However, PET studies demonstrating decreased cortical binding of GABAergic, serotonergic, and now dopaminergic ligands in ALS patients argues against dopamine-specific pathogenesis of ALS and supports a generalized cortical neuronal loss or dysfunction in disease.

### Oxidative Stress

Oxidative stress is considered one of the pathogenic mechanisms underlying neurodegeneration in ALS (76) and is the proposed target of edaravone, a free radical scavenger recently approved for treatment of ALS (77). The PET ligand [^62^Cu]-ATSM is a copper-linked small molecule structurally similar to superoxide dismutase (78). It distributes to areas of hypoxia and oxidative stress in PET studies of patients with Parkinson's disease (79) and mitochondrial diseases (80). One [^62^Cu]-ATSM PET study in ALS showed increased tracer accumulation in the motor cortices, paracentral lobules, and right superior parietal lobule in ALS patients compared to controls (63). Areas of increased uptake negatively correlated with ALSFRS-R score.

Notably, Cu-ATSM was selected as an investigational drug for ALS because human [^62^Cu]-ATSM PET studies demonstrated effective penetration into the brain. Cu-ATSM's proposed mechanism of action is free radical scavenging and delivery of copper into the CNS (81). Cu-ATSM slowed disease progression in SOD^G93A^ mouse models of ALS (81, 82) and is now entering phase I human clinical trials for ALS (Clinicaltrials.gov NCT02870634).

## Challenges and Opportunities in the Development of Molecular Imaging Biomarkers for ALS

The FDA-NIH Biomarker Working Group defines a biomarker as a “characteristic that is measured as an indicator of normal biological processes, pathogenic processes, or responses to an exposure or intervention, including therapeutic interventions” (83). Because PET imaging can localize molecular changes in the brain, it has unique promise for use as a diagnostic, prognostic, and pharmacodynamic biomarker. Its advantages and disadvantages complement other biomarkers being developed for ALS (Table 2).

**Table 2:** Advantages and disadvantages of potential biomarkers for ALS.

**Biomarker type**	**Advantages**	**Disadvantages**
**Neuroimaging biomarkers:** Positron emission tomography (PET)	Ability to interrogate disease mechanisms of interest using specific molecular ligands (e.g., energy metabolism, neuroinflammation, neuronal dysfunction, oxidative stress) Dynamic ligand binding enables visualization of treatment effect in central nervous system (CNS) Sensitive to early pathological changes Localizes pathology in CNS	Cost Limited scalability due to expertise and resources required (local cyclotron for production of radioisotopes) Small risk associated with repeated radiation Use may be limited by patient orthopnea
**Neuroimaging biomarkers:** Magnetic resonance imaging (MRI)	Widely available Advanced techniques permit evaluation of brain activation (functional MRI), white matter tracts (diffusion tensor imaging), and cellular metabolites (magnetic resonance spectroscopy) Free of radiation Localizes pathology in CNS	Large sample sizes required to demonstrate treatment effect limits pharmacodynamic potential (84) Use may be limited by patient orthopnea
**Biological fluid-based biomarkers**	Scalable Cost-effective Ease of collection Potential for standardization and centralization in core laboratory	Non-localizing
**Electrophysiological biomarkers**	Directly measures physiology of organs affected by disease Accepted use in diagnosis (electromyography) Sensitive to early pathological changes Good face value for monitoring disease progression	Reliability and reproducibility Sensitive to technical artifacts Potential patient discomfort (electromyography)

### PET Imaging as a Diagnostic Biomarker

Mounting evidence of quantifiable PET imaging differences between ALS and control brains has generated interest in using PET as a diagnostic biomarker for ALS. Indeed, the sensitivity of PET makes it uniquely positioned to detect or confirm UMN dysfunction in suspected ALS patients, which has traditionally been difficult to measure.

Three successive studies recently assessed the diagnostic potential of [^18^F]-FDG PET in ALS (17, 32, 39). In these studies, the authors used group differences in scans from ALS and control subjects to generate algorithms (“diagnostic algorithms”) for classifying individual scans as ALS vs. control. Group-level differences in FDG uptake between ALS and control scans were consistent across time and between two imaging centers. Within one center, the diagnostic algorithm generated from a training cohort achieved high accuracy when classifying scans from a validation cohort (as ALS or control), though accuracy decreased when scans from PLS patients were included in the analysis (17). However, in a multicenter study, the diagnostic algorithm derived from one center's scans (training cohort) achieved 94.8% sensitivity but only 12.5% specificity in classifying scans from a second center (validation cohort) as ALS or control (39). The low specificity was attributed to relative frontal hypometabolism in the validation control scans, compared to training control scans. The validation control scans came from patients with non-neurologic malignancies undergoing surveillance brain PET, whereas the training control scans came from healthy volunteers.

These studies highlight the challenges in translating population-level PET data into diagnostic criteria for individual patients. While progress is being made, PET is not yet a valid diagnostic biomarker for ALS. Validation will require longitudinal studies to determine whether prospectively collected scans of patients undergoing evaluation for ALS can distinguish UMN dysfunction before clinical signs emerge. The studies will also need to distinguish motor neuron disease not just from healthy volunteers, but also from disease mimics. If validated as a diagnostic biomarker, PET imaging could shorten the time from ALS symptom onset to diagnosis and facilitate earlier intervention in the neurodegenerative process.

### PET Imaging as a Prognostic Biomarker

PET imaging has potential for prognostic use in ALS. Two studies in ALS patients found an association between mortality rate and presence of extensive frontotemporal hypometabolism on [^18^F]-FDG PET scans (17, 32). Conversely, patients with spinal cord hypermetabolism in the top 20% of one study cohort had a significantly higher mortality rate compared to the rest of the cohort (36). Further longitudinal studies that evaluate whether PET imaging findings can predict the likelihood of future events (such as survival, development of cognitive impairment, or spread of disease from one anatomical region to another) are needed to establish valid prognostic PET biomarkers in ALS.

One intriguing potential use for prognostic PET imaging is in identifying when asymptomatic ALS gene carriers enter a high-risk period for developing clinical disease (“phenoconversion”). Rising levels of serum neurofilament light chain can detect neurodegeneration ~1 year before phenoconversion in asymptomatic ALS gene mutation carriers (13). To evaluate whether PET imaging can detect also changes that predict phenoconversion, longitudinal [^11^C]-PBR28 PET studies are being conducted in asymptomatic gene mutation carriers to look for neuroinflammation before disease onset. Prognostic biomarkers of phenoconversion may facilitate development of gene therapy trials designed to prevent ALS, which may be the best opportunity for treating or even curing certain genetic forms of ALS.

### PET Imaging as a Pharmacodynamic Biomarker

PET imaging has value as a pharmacodynamic marker in ALS because it can rapidly measure and localize biological activity of investigational agents in the target tissue—the brain. The variety of available PET ligands may enable direct visualization of multiple pharmacologic targets. PET imaging's sensitivity to molecular changes can increase statistical power to detect a drug effect.

[^11^C]-PBR28 PET is an appealing pharmacodynamic biomarker for ALS clinical trials because binding is dynamic and rapidly responsive to treatment: in Parkinson's disease (85) and traumatic brain injury (86) patients, anti-inflammatory treatment reduced cortical [^11^C]-PBR28 binding in as little as 4 weeks (85). Additionally, the stability of [^11^C]-PBR28 uptake in ALS over 6 months of disease progression permits a marked reduction in sample size needed to determine drug effect. A simulated sample size and power calculation using longitudinal [^11^C]-PBR28 PET data found that 30 participants are needed in a single-arm ALS clinical trial to show a 2% change in [^11^C]-PBR28 uptake after drug treatment, whereas hundreds of participants are needed to show a 30% reduction in ALSFRS-R slope (50). Currently, four ongoing clinical trials are using [^11^C]-PBR28 PET as a pharmacodynamic biomarker to assess the biological activity of investigational treatments in ALS (Clinicaltrials.gov NCT02714036, NCT02469896, NCT03127514, NCT03456882) (87).

In the future, PET imaging using an array of ligands will enable efficient evaluation of multiple pharmacologic targets. Pharmacodynamic data from PET studies may help confirm the biological activity of ALS drugs in the target tissue and inform dose selection based on biological activity. Data derived from these trials will enable deeper understanding of the role of different molecular mechanisms in disease pathogenesis.

## Conclusions and Future Directions

Thirty years of PET imaging has shed light on the pathophysiology of ALS and the expanding boundaries of cortical dysfunction in disease. Because PET imaging can localize molecular changes in the CNS *in vivo*, it has the potential to fill a critical gap in our armamentarium of diagnostic, prognostic, and pharmacodynamic biomarkers for ALS. To realize this potential, major limitations of the research to date will need to be addressed. First, most PET studies in ALS were small. Only 7 published studies enrolled more than 50 ALS patients (17, 31–33, 35, 39, 50), which raises concern for false positive and/or negative findings generated by studies with small sample sizes. Second, minimal longitudinal PET data exists in the ALS literature. A total of 24 ALS patients have had longitudinal PET scans in published studies (18, 22, 24, 27, 45, 50). Third, clinical-radiological correlations reported in the literature are insufficiently characterized and often contradictory. To address these limitations, we must conduct collaborative, multicenter longitudinal studies to collect PET imaging and clinical data in large patient cohorts. Moreover, to ascertain accurate clinical-radiologic correlations, clinical data should be captured by validated instruments that separate motor and cognitive deficits and reliably measure UMN dysfunction.

From a practical standpoint, the widespread use of PET imaging is presently limited by cost, need for expertise and local production of radioactive isotopes. Therefore, PET imaging currently is most useful as a pharmacodynamic biomarker for early clinical trials in ALS. Future multicenter longitudinal studies will allow us to establish the relationship between PET imaging findings and meaningful clinical outcomes, and thus develop and validate the PET imaging biomarkers that can accelerate drug development and advance care for people with ALS.

## Disclosure

NA receives consulting fees from Biogen Idec and MT Pharma.

## Author Contributions

SC and NA drafted the manuscript. Both authors made a direct and intellectual contribution to the work and approved it for publication.

### Conflict of Interest Statement

The authors declare that the research was conducted in the absence of any commercial or financial relationships that could be construed as a potential conflict of interest.
